# Type I interferon suppresses tumor growth through activating the STAT3-granzyme B pathway in tumor-infiltrating cytotoxic T lymphocytes

**DOI:** 10.1186/s40425-019-0635-8

**Published:** 2019-06-22

**Authors:** Chunwan Lu, John D. Klement, Mohammed L. Ibrahim, Wei Xiao, Priscilla S. Redd, Asha Nayak-Kapoor, Gang Zhou, Kebin Liu

**Affiliations:** 1 Department of Biochemistry and Molecular Biology, Medical College of Georgia, Augusta, GA 30912 USA; 2 Georgia Cancer Center, Medical College of Georgia, Augusta, GA 30912 USA; 3 Charlie Norwood VA Medical Center, Augusta, GA 30904 USA

**Keywords:** Type I interferon, CTLs, STAT3, Granzyme B, Colon Cancer

## Abstract

**Background:**

Type I interferons (IFN-I) have recently emerged as key regulators of tumor response to chemotherapy and immunotherapy. However, IFN-I function in cytotoxic T lymphocytes (CTLs) in the tumor microenvironment is largely unknown.

**Methods:**

Tumor tissues and CTLs of human colorectal cancer patients were analyzed for interferon (alpha and beta) receptor 1 (IFNAR1) expression. IFNAR1 knock out (IFNAR-KO), mixed wild type (WT) and IFNAR1-KO bone marrow chimera mice, and mice with IFNAR1 deficiency only in T cells (IFNAR1-TKO) were used to determine IFN-I function in T cells in tumor suppression. IFN-I target genes in tumor-infiltrating and antigen-specific CTLs were identified and functionally analyzed.

**Results:**

IFNAR1 expression level is significantly lower in human colorectal carcinoma tissue than in normal colon tissue. IFNAR1 protein is also significantly lower on CTLs from colorectal cancer patients than those from healthy donors. Although IFNAR1-KO mice exhibited increased susceptibility to methylcholanthrene-induced sarcoma, IFNAR1-sufficient tumors also grow significantly faster in IFNAR1-KO mice and in mice with IFNAR1 deficiency only in T cells (IFNAR1-TKO), suggesting that IFN-I functions in T cells to enhance host cancer immunosurveillance. Strikingly, tumor-infiltrating CTL levels are similar between tumor-bearing WT and IFNAR1-KO mice. Competitive reconstitution of mixed WT and IFNAR1-KO bone marrow chimera mice further determined that IFNAR1-deficient naïve CTLs exhibit no deficiency in response to vaccination to generate antigen-specific CTLs as compared to WT CTLs. Gene expression profiling determined that *Gzmb* expression is down-regulated in tumor-infiltrating CTLs of IFNAR1-KO mice as compared to WT mice, and in antigen-specific IFNAR1-KO CTLs as compared to WT CTLs in vivo. Mechanistically, we determined that IFN-I activates STAT3 that binds to the *Gzmb* promoter to activate *Gzmb* transcription in CTLs.

**Conclusion:**

IFN-I induces STAT3 activation to activate *Gzmb* expression to enhance CTL effector function to suppress tumor development. Human colorectal carcinoma may use down-regulation of IFNAR1 on CTLs to suppress CTL effector function to evade host cancer immunosurveillance.

**Electronic supplementary material:**

The online version of this article (10.1186/s40425-019-0635-8) contains supplementary material, which is available to authorized users.

## Background

Type I interferons IFNα and IFNβ (IFN-I) are pleiotropic cytokines that were originally identified as viral replication suppressor. However, IFN-I function has since been extended to cancer suppression [1–5] and IFNα is now approved for the treatment of both solid and hematologic tumors [6–8]. Unlike the type II interferon IFNγ, which exhibits restricted expression in activated T cells and NK cells [9], there are 14 isoforms of IFNα [10] that are expressed in hematopoietic cells, particularly in dendritic cells (DCs), whereas IFNβ is expressed in most cell types [11]. The mechanism underlying IFN-I function in tumor suppression is currently an extensively studied subject and it has long been thought that IFN-I suppresses tumor development through their intrinsic functions in tumor cells. Indeed, IFN-I regulates the expression of various genes that modulate tumor cell growth, proliferation, migration, apoptosis, tumor antigen expression, and immune checkpoint-mediated immune suppression [12–18]. In human cancer patients, the tumor cell autologous IFN-I signaling controls cancer response to chemotherapy [1], and mutations in mediators of the IFN-I signaling pathways in tumor cells confer human cancer non-response to immune checkpoint inhibitor immunotherapy [19, 20].

Recent breakthroughs in immune checkpoint inhibitor cancer immunotherapy demonstrated the critical role of activated T cells in host cancer immunosurveillance. T cells are the main adaptive immune cells that directly target tumor cells for lysis [21–23]. It is well documented that IFN-I deficiency leads to increased tumor incidence [24] and IFN-I regulates dendritic cell priming of T cells to execute tumor suppressive activity [25]. Therefore, in addition to the tumor cells, T cell might be another target of IFN-I in regulation of anti-tumor immune response. However, the intrinsic function of IFN-I in regulating T cell activation and cytotoxicity in the context of host cancer immunosurveillance is largely unknown. We report here that the intrinsic IFN-I signaling pathway is essential for CTL effector function in tumor suppression and human colorectal carcinoma may use down-regulation of the IFNAR1 on CTLs to impair CTL effector function to evade host cancer immunosurveillance. Our findings thus extend IFN-I function to CTLs in host cancer immunosurveillance.

## Methods

### Mice and human specimens

IFNAR1 knock out mice [B6(Cg)-*Ifnar1*^*tm1.2Ees*^/J (IFNAR1-KO) were obtained from Jackson Laboratory (Bar Harbor ME). Mice with IFNAR1 deficiency only in T cells (IFNAR1-TKO) were created by crossing *Ifnar1*^*fl*^ (B6(Cg)-*Ifnar1*^*tm1.1Ees*^/J) mouse with B6.Cg-Tg (*lck-cre*)548Jxm/J mouse (Jackson Laboratory). SJL (B6.SJL-*Ptprc*^*a*^ *Pepc*^*b*^/BoyJ) and female C57BL/6 mice were obtained from the Jackson Laboratory. The control and experiment groups are age and sex-matched mice. Use of mice was performed according to approved protocols by institutional animal use and care committee. Peripheral blood samples were collected from consented healthy donors in Shepheard Community Blood bank. Human colon cancer patient blood specimens were collected from consented patients in Georgia Cancer Center under approved protocol by Augusta University Institutional Review Board.

### Mouse tumor models

Sarcoma was induced by injecting methylcholanthrene (MCA, 100 μg/mouse in peanut oil, Sigma-Aldrich, St Louis, MO) to mice subcutaneously.

### Mixed bone marrow chimera mouse model and immunizations

Mixed BM chimera mice was created as previously described [26] using BM cells from SJL and IFNAR1-KO mice (at 1:1 ratio of SJL: IFNAR1-KO) (Additional file 1 Figure S2). Mice were immunized with the 2W1S peptide (EAWGALANWAVDSA) to activate CD4^+^ T cells [27] and with the OVA peptide (SIINFEKL) to activate CD8^+^ T cells [28] as previously described and analyzed for antigen-specific T cells as previously described [26].

### Tumor cell lines

Murine colon carcinoma MC38 cells were characterized as previously described [29].

### Antibodies and reagents

Fluorescent dye-conjugated antibodies that are specific for CD45, CD4, CD8, and Zombie violet were obtained from Biolegend (San Diego, CA). pSTAT1 inhibitor Fludarabine [30] and pSTAT3 inhibitor Stattic [31] were obtained from Santa Cruz. The 2W1S and OVA tetramers were provided by the NIH Tetramer Core Facility (Emory University, GA). The cells were stained with 0.15 μl 2W1S and 0.25 μl OVA tetramers. All the antibodies and reagents are listed in Additional file 1 Table S1.

### Analysis of DNA-protein interactions by electrophoretic mobility shift assay (EMSA)

Tumor-specific 2/20 CTLs were maintained as previously described [32]. T cells were cultured with recombinant IFNα, and IFNβ, respectively for 1 h for nuclear extract preparation. The WT pSTAT3 consensus probe forward sequence is 5′- GATCCTTCTGGGAATTCCTAGATC − 3′ and reverse sequence is 3′- CTAGGAAGACCCTTAAGGATCTAG-5′ (Santa Cruz Cat# sc-2571). The pSTAT3 mutant probe forward sequence is 5′- GATCCTTCTGGGCCGTCCTAGATC-3′ and reverse sequence is 3′-CTAGGAAGACCCGGCAGGATCTAG-5′ (Santa Cruz cat# sc-2572). The end-labeled pSTAT3 probe were incubated with nuclear extracts and analyzed by EMSA as previously described [33].

### Gene expression and Western blotting analysis

Gene expression was analyzed using RNA and gene-specific primers in the StepOne Plus Real-Time PCR System (Applied Biosystems). The PCR primers are: mouse *Gzmb* forward 5′- GCCCACAACATCAAAGAACAGG-3′, Gzmb reverse 5′-CGTATCAGGAAGCCACCGCAC-3′; mouse β-actin forward 5′- TGAAGGTGACAGCAGTCGGTTG-3′, β-actin reverse 5′- GGCTTTTAGGATGGCAAGGGAC-3′. Western blotting analysis was performed as previously described [34]. Antibodies are listed in Additional file 1 Table S1.

### Analysis of immune gene expression in CTLs

Tumor tissues were digested with collagenase, followed by incubation with anti-CD8 mAb-coated magnetic beads (Biolegend), and separation by a magnetic stand. RNA was purified from cells bound to the beads. WT and IFNAR1-KO CD8^+^ T cells were also isolated from OVA peptide-vaccinated mice by cell sorting and used for RNA purification. RNA was hybridized overnight with reporter and capture code set using the Nanostring immunology gene panel at 65 °C and analyzed on an nCounter instrument according to the manufacturer’s instructions. Digital images are processed within the nCounter instrument, and the Reporter Probe counts were tabulated in a comma separated value (CSV) format for convenient data analysis with NanoString’s free nSolver™ Analysis Software V.3.

### Statistical analysis

All statistical analysis were performed by two-sided Student *t* test using the GraphPad Prism program (GraphPad Software, Inc.). *p* < 0.05 is considered as statistically significant.

## Results

### IFNAR1 is down-regulated in CTLs of human colon cancer patients

**Fig. 1:**
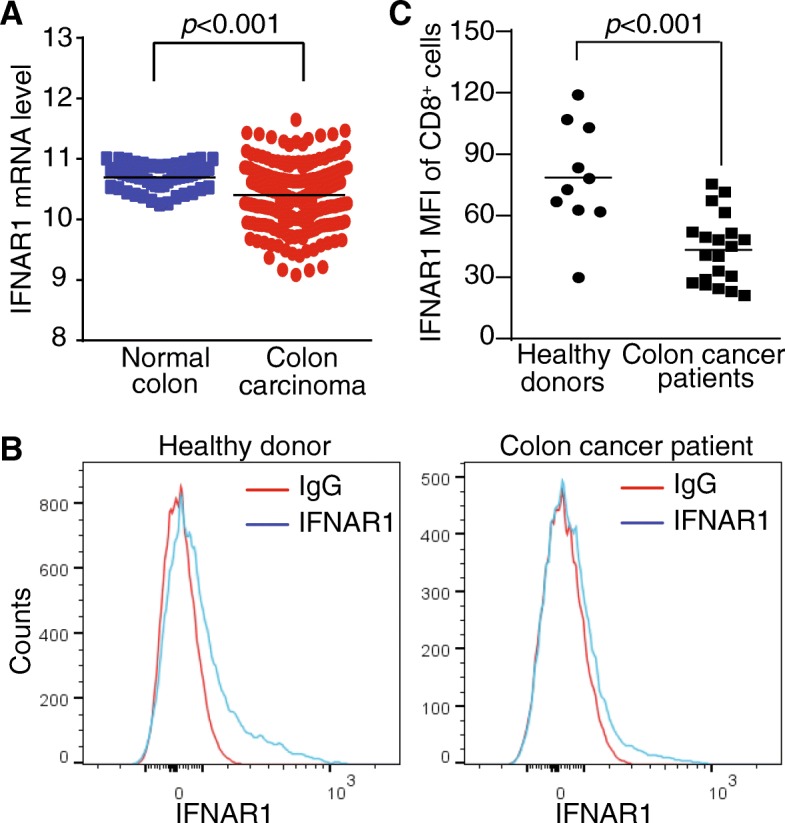
IFNAR1 is down-regulated in CTLs of human colon cancer patients. **a**. IFNAR1 mRNA expression level data was extracted from TCGA Colon Cancer (COAD) dataset using GEPIA Gene Expression Profiling Interactive Analysis (GEPIA) Cancer Genomics Browser. The IFNAR1 expression level between human colon carcinoma tissues (*n* = 380) and adjacent normal tissues (*n* = 51) were compared. **b**. PBMCs were isolated from peripheral blood specimens of healthy donors (*n* = 10) and colon cancer patients (*n* = 20), stained with CD8- and IFNAR1-specific antibodies, and analyzed by flow cytometry. CD8^+^ T cells were gated for IFNAR1 receptor level (MFI). Shown are representative IFNAR1 MFI histograms of CD8^+^ T cells from one healthy donor (left panel) and one colon cancer patient (right panel). Staining with IgG isotype antibody was used as negative control. **c**. Quantification of CD8^+^ T cell IFNAR1 MFIs of healthy donors and cancer patients. Each dot represents IFNAR1 MFI of one donor or patient

IFNAR1 mediates all isoforms of IFN-I signaling. Analysis of TCGA dataset revealed that the IFNAR1 expression level is significantly down-regulated in human colon carcinomas as compared to the normal colon tissues (Fig. 1A). The tumor tissue is a mixture of tumor cells and immune cells, we then compared IFNAR1 protein level on CD8 ^+^ T cells from healthy donors and colon cancer patients. The IFNAR1 protein level is significantly lower on CD8^+^ T cells from human colon cancer patients as compared to that from healthy donors (Fig. 1B&1C). These findings indicate that CTLs in human colon cancer patients are deficient in IFN-I signaling.

### IFN-Ι suppresses tumor development through a T cell-dependent mechanism

**Fig. 2:**
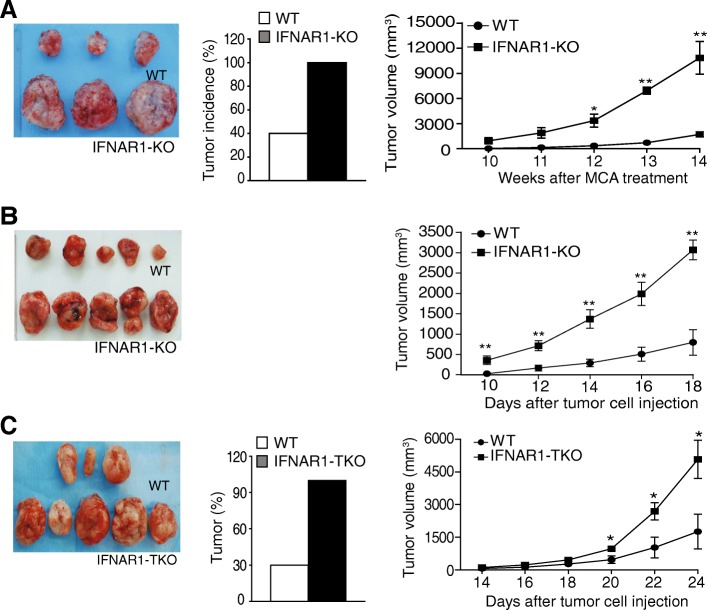
IFN-I suppresses tumor growth via an immune cell-dependent mechanism. **a**. WT (*n* = 10) and IFNAR1-KO mice (*n* = 5) were injected with MCA at the right flank (100 mg/mouse in peanut oil). Tumor growth was monitored over time starting at week 10. Two IFNAR1-KO mice developed tumor at 56 days after MCA injection and were sacrificed on day 86 due to tumor size limitation of the animal use protocol before the end of the experiment. Only 4 of the 10 WT mice developed tumor. Three of the WT and three of the IFNAR1-KO mice developed tumor around 10 weeks after MCA injection. One WT mouse developed tumor 90 days after MCA injection. Shown are tumor images from the three pairs of WT and IFNAR1-KO mice that developed tumor at about the same time (left panel). Tumor incidence is presented at the middle panel. Tumor growth kinetics in the three pairs of WT and IFNAR1-KO mice as shown in the right panel. * *p* < 0.05. ** *p* < 0.01. **b**. Murine colon carcinoma MC38 cells were injected to WT (*n* = 5) and IFNAR1-KO (*n* = 5) mice. Mice were monitored for tumor size starting at day 10 and sacrificed 18 days after tumor cell injection. Shown are tumor images (left panel) and tumor growth kinetics (right panel). ** *p* < 0.01. **c**. MC38 cells were injected s.c. to WT mice (*n* = 10) and mice with IFNAR1 deficiency only in T cells (IFNAR1-TKO, *n* = 5). Tumor formed in 3 of the 10 WT and 5 of the 5 IFNAR1-TKO mice. Shown are tumor image (left panel) and tumor growth incidence (middle panel). Tumor growth kinetics of the tumors as shown in the right panel. * *p* < 0.05

Because IFNAR1 mediates all isoforms of IFN-I signaling, our above findings suggest that human colon carcinoma might use down-regulating IFNAR1 to impair IFN-I signaling in CTLs to evade immune surveillance. To determine IFN-I function in CTLs in anti-tumor immune response, we sought to determine IFN-I function in tumor development. WT and IFNAR1-KO mice were injected with MCA and monitored for tumor development. About 40% of WT mice developed tumor 14 weeks after MCA injection. In contrast, all IFNAR1-KO mice developed tumors (Fig. 2A). Furthermore, the IFNAR1-KO tumor grew significantly faster than the WT tumor from 12 weeks to 14 weeks (Fig. 2A). To determine IFN-I function in the immune component of the tumor microenvironment, The IFNAR1 sufficient MC38 tumor cells were then transplanted to WT and IFNAR1-KO mice. In this model, only host immune cells are deficient in IFNAR1. As in the WT and IFNAR1-KO tumor-bearing mice, the MC38 tumor grew significantly faster in the IFNAR1-KO mice than in the WT mice from 10 days to 18 days after tumor injection (Fig. 2B). These findings indicate that IFN-I suppresses tumor development at least in part through an immune cell-dependent mechanism.

To determine whether IFN-I acts in T cells to suppress tumor development, we next created mice with IFNAR1 deletion only in T cells (IFNAR1-TKO). IFNAR1-TKO mice showed no differences in T cell profiles as compared to WT mice, and NK cell number is significantly higher in IFNAR1-TKO mice as compared to WT mice, albeit at a small degree (Additional file 1 Figure S1). MC38 tumor cells were transplanted to WT and IFNAR1-TKO mice. MC38 cells formed tumor in about 30% of the WT mice. In contrast, tumor formed in all IFNAR1-TKO mice (Fig. 2C). Furthermore, the established tumor grew significantly faster and bigger in IFNAR1-TKO mice as compared to the WT mice (Fig. 2C). These findings thereby indicate that IFN-I suppresses tumor growth at least in part through regulating T cell function in the tumor microenvironment.

### Immune cell profiles in the tumor-bearing mice

**Fig. 3:**
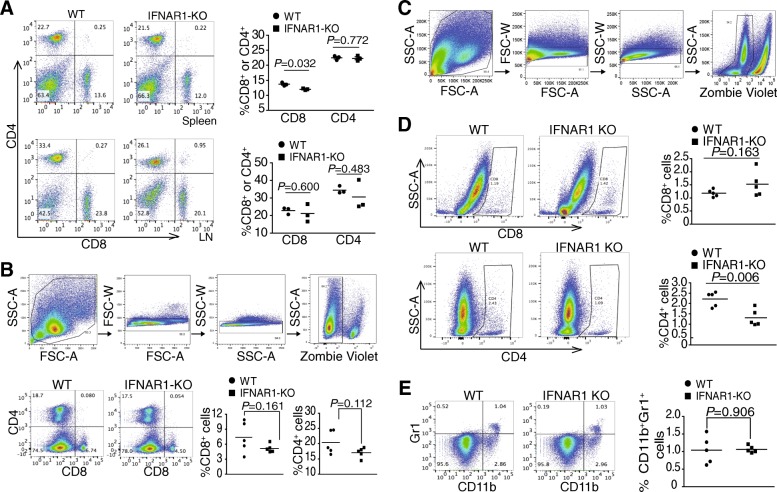
Type I interferon and immune cell profiles in the tumor microenvironment. **a.** Spleen cells from tumor-free mice were stained with CD4- and CD8-specific mAbs and analyzed by flow cytometry. Shown are representative flow cytometry plots. The % CD4^+^ and CD8^+^ T cells were quantified and presented at the right panel. **b**. Cells were prepared from spleens of the MC38 tumor-bearing WT (*n* = 5) and IFNAR1-KO (*n* = 5) mice as shown in Fig. 2B. Top panel shows gating strategy for spleen cells. Single cells were gated out for live and dead cells. The live cells were gated for CD4^+^ and CD8^+^ T cells as in A. **c**. Gating strategy for tumor-infiltrating immune cells. Tumor were excised from the MC38 tumor-bearing WT (n = 5) and IFNAR1-KO (n = 5) mice, digested with collagenase to make single cells. The digested tumor cell mixtures were stained with Zombie violet and CD4-, CD8-, CD11b-, and Gr1-specific mAbs. The digested cells were gated for single cells, followed by gating for live and dead cells. **d** & **e**. The live cells as shown in C were analyzed for T cells (**d**) and CD11b^+^Gr1^+^ cells (**e**) by flow cytometry. Shown at the left panels are representative plots of one pair of mice. The % tumor-infiltrating CD4^+^ CD8^+^ T cells (**d**) and CD11b^+^Gr1^+^ cells (**e**) were quantified and presented at the right panel

We next analyzed T cells in the MC38 colon tumor model as shown in Fig. 2B. There is small difference in spleen CD8^+^ T cells between tumor-free WT and IFNAR1-KO mice. No significant difference in lymph node and spleen CD4^+^ and no significant difference in lymph node CD8^+^ T cell levels between the tumor-free WT and IFNAR1-KO mice were observed (Fig. 3A). There is also no significant difference in CD4^+^ and CD8^+^ T cell levels in the spleens of tumor-bearing WT and IFNAR1-KO mice (Fig. 3B). The tumor-infiltrating CD4^+^ T cell levels decreased significantly in the tumor-bearing IFNAR1-KO mice as compared to the WT tumor-bearing mice (Fig. 3C & D). However, there is no significant difference in tumor-infiltrating CD8^+^ T cell level between the WT and IFNAR1-KO mice (Fig. 3C & D). No significant difference was observed in tumor-infiltrating CD11b^+^Gr1^+^ MDSCs levels between WT and IFNAR1-KO mice (Fig. 3C & E). These observations indicate that IFN-I play no essential role in CTL tumor infiltration and differentiation.

### IFN-I and antigen-specific T cell activation and differentiation

**Fig. 4:**
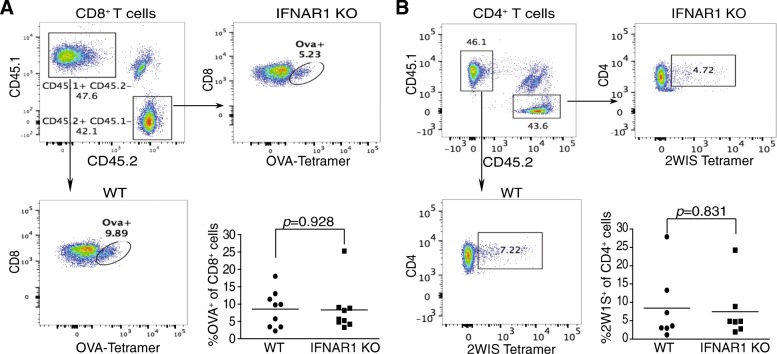
IFN-I regulation of T cell activation in vivo. **a**. Mixed WT and IFNAR1 chimera mice were vaccinated sixty nine days after BM adoptive transfer to induce OVA-specific CD8^+^ T cells. The mice received a prime followed by a boost 14 days later. Blood samples were collected and analyzed seven days following the boost. Shown is the analysis gating strategy of white blood cells. The levels of OVA-specific WT and IFNAR1-KO CD8^+^ T cells were quantified and presented at the bottom right panel. Each dot represents data from one mouse. **b**. The mixed chimera mice were vaccinated sixty nine days after BM adoptive transfer with 2W1S peptide to induce CD4-specific activation. 2W1S-specific WT and IFNAR1-KO CD4^+^ T cells were analyzed and quantified as in A

The IFNAR1-TKO mice showed significant deficiency in tumor growth control as compared to WT mice (Fig. 2C). It is therefore unexpected that deficiency in IFNAR1 does not lead to altered CTL tumor infiltration and differentiation (Fig. 3C & D). To further determine IFN-I function in T cells and to unmask the effects of IFN-I on T cells from IFN-I-deficiency-related DC deficiency [25], we created mixed bone marrow chimera mice with WT and IFNAR1-KO mice. The mixed chimera mice have WT DCs from WT BM and thus have functional DC to let us determine the direct function of IFN-I in WT and IFNAR1-KO CD4^+^ and CD8^+^ T cells within the same cellular microenvironment. We used two vaccination models [27, 28] to stimulate antigen-specific CD4^+^ and CD8^+^ T cell responses in the mixed chimera mice in vivo. The WT (CD45.1^+^) and IFNAR1-KO (CD45.2^+^) CD4^+^ and CD8^+^ T cells were then determined. Interestingly, both CD4^+^ and CD8^+^ IFNAR1-KO T cells were as responsive to antigen stimulation as WT T cells in the mixed chimera mice. As observed in the tumor-bearing mice, there are no significant differences in the levels of antigen-specific CD8^+^ T cells (Fig. 4A). Although CD4^+^ tumor-infiltrating T cells were significantly lower in IFNAR1-KO tumor-bearing mice as compared to WT tumor-bearing mice (Fig. 3C & D), IFNAR1-deficient CD4^+^ naïve T cells responded to antigen stimulation to generate antigen-specific CD4^+^ T cells as efficiently as WT CD4^+^ T cells in the mixed chimera mice (Fig. 4B). We therefore conclude that IFN-I is not essential for antigen-specific T cell activation and differentiation in vivo.

### IFN-I regulation of granzyme B expression in CTLs

**Fig. 5:**
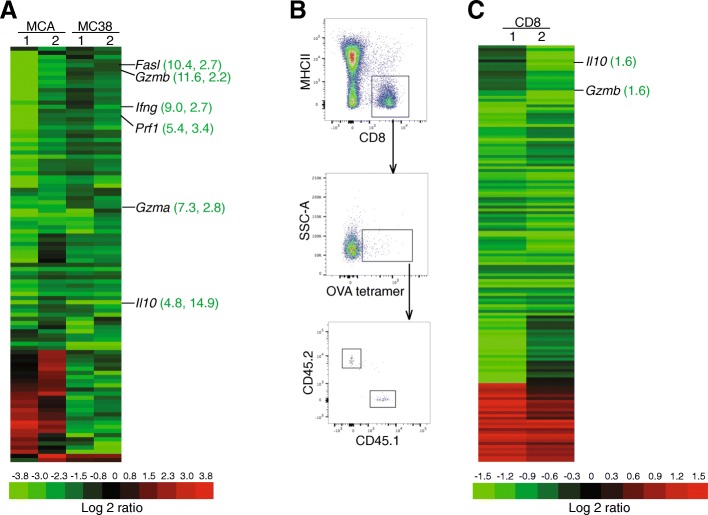
IFN-I regulates expression of granzyme B in tumor-infiltrating and antigen-specific CTLs. **a**. RNA was isolated from tumor-infiltrating CTLs from MC38 (18 days after tumor injection) and MCA (96 days after MCA injection) tumor models as outlined in Fig. 2A and B and analyzed for gene expression using the Nanostring immunology gene panel. Genes whose expression levels are 2 or more folds different in tumor-infiltrating CTLs between WT and IFNAR1-KO mice were clustered and presented. Green color indicates higher in WT and red color indicates lower in WT mice. The numbers in the parentheses represent fold decrease in IFNAR-KO mice as compared to WT mice. **b**. Spleen cells from the WT and IFNAR1-KO mixed BM chimera mice were collected 14 days after boost and stained with MHCII-, CD8-, CD45.1-, CD45.2-specific mAbs and OVA tetramer. The activated (OVA tetramer-positive) WT (CD45.1^+^) and IFNAR1-KO (CD45.2^+^) CD8^+^ cells were gated as indicated and sorted for mRNA purification. **c**. RNAs were prepared from sorted cells as shown in B. Fifty ng RNA were analyzed for gene expression using the Nanostring immunology gene panel. Genes whose expression levels are 1.5 or more folds different between activated WT and IFNAR1-KO CD8^+^ T cells from the mixed chimera mice as shown in B were clustered and presented

The above findings strongly suggest that IFN-I regulates CTL effector function rather than regulating T cell activation to execute its anti-tumor activity. To test this hypothesis, we isolated tumor-infiltrating CD8^+^ CTLs from the total tumor tissues of the WT and IFNAR1-KO MCA (Fig. 2A) and MC38 (Fig. 2B) tumor-bearing mice and analyzed expression profiles of immune genes (Fig. 5A). Interestingly, the expression levels of five key CTL effector genes, *Fasl*, *prf1*, *Gzma*, *Gzmb* and *Ifng*, and the immune suppressive gene *Il10*, were significantly down-regulated in the IFNAR1-KO tumor-infiltrating CTLs from both MCA and MC38 tumor, respectively, as compared to the WT tumor-infiltrating CTLs (Fig. 5A). The list of all differentially expressed genes is presented in Additional file 1 Table S2. The Fas-FasL pathway and the perforin-granzyme B pathway are the two primary effector mechanisms that CTLs use to kill target cells [35, 36]. To determine whether IFN-I regulation of FasL and perforin/granzyme B expression is a general phenomenon in CTLs, a complimentary approach was then used to validate this finding, we sorted antigen-specific WT and IFNAR1-KO CD8^+^ CTLs from spleens of the OVA vaccinated mixed chimera mice (Fig. 5B). The cells were then analyzed for the expression of immune genes. *Gzmb* and *Il10* expression levels decreased 1.6 folds in the IFNAR1-KO OVA-specific CTLs as compared to the WT OVA-specific CTLs (Fig. 5C). The list of all differentially expressed genes is presented in Additional file 1 Table S3. These observations indicate that IFN-I is a general regulator of CTL effector granzyme B expression.

### IFN-I induces STAT3 activation to activate *Gzmb* transcription

**Fig. 6:**
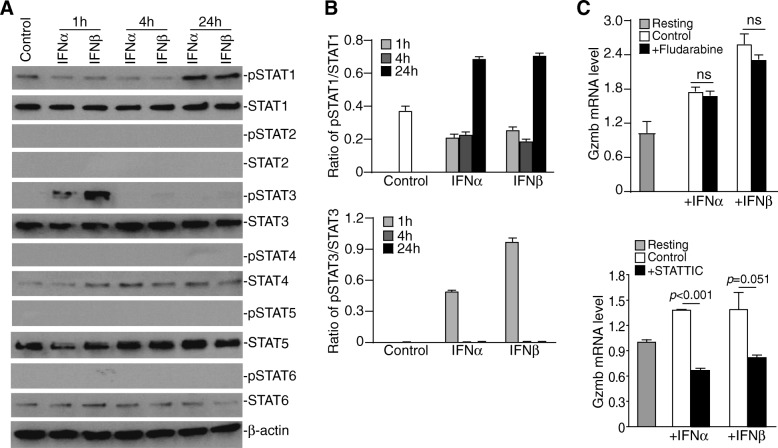
IFNα and IFNβ activate STAT3 to up-regulate *Gzmb* expression in CTLs. **a**. the tumor-specific resting 2/20 CTLs were cultured in the presence of IFNα and IFNβ, respectively, and analyzed by Western blotting analysis for the indicated STATs. **b**. The protein band intensities of pSTAT1 and pSTAT3 as shown in A were quantified using NIH image J and normalized as the ratio over the intensities of STAT1 and STAT3, respectively. Column: Mean; Bar: SD. **c**. Resting 2/20 CTLs were treated with recombinant IFNα and IFNβ, respectively, in the absence (control) or presence of pSTAT1 (+Fludarabine, 10 μM, top panel) and pSTAT3 (+STATTIC, 5 μM, bottom panel) inhibitors, respectively, for 24 h. Cells were analyzed by qPCR for *Gzmb* expression level

We next used a defined CTL system to determine the function of IFN-I in regulation of granzyme B expression. 2/20 CTLs is an H-2L^d^-restricted tumor cell-reactive CTL line that recognizes the AH1 peptide of the gp70 viral protein [37]. We first treated 2/20 CTLs with IFNα and IFNβ and analyzed STAT activation. STAT1, STAT3, STAT4, STAT5 and STAT6 were detectable in the resting CTLs and only total STAT4 protein level was increased by IFNα and IFNβ treatment. Treatment of resting 2/20 CTLs with IFNα and IFNβ induced STAT3 activation at 1 h and STAT1 activation at 24 h, respectively, after treatment (Fig. 6A & B). CTLs were then treated with IFNα and IFNβ in the presence of pSTAT1-specific [30] and pSTAT3-specific [31] inhibitor, respectively. Analysis of granzyme B expression revealed that inhibition of pSTAT1 does not cause significant change in granzyme B expression, but inhibition of STAT3 activation diminished granzyme B expression up-regulation induced by IFNα and IFNβ (Fig. 6C). We therefore conclude that IFN-I induces STAT3 to activate *Gzmb* expression in CTLs.

**Fig. 7:**
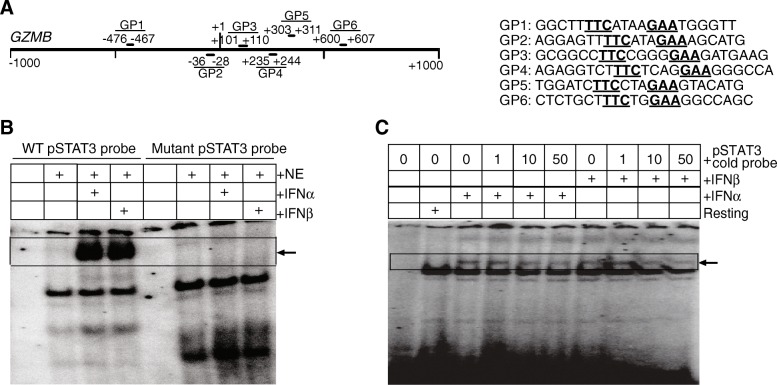
IFNα and IFNβ-activated STAT3 binds to the *Gzmb* promoter in CTLs. **a**. Structures of the *Gzmb* promoter. The six putative ISRE sequences (right panel) and locations (left panel) are shown. **b**. Resting 2/20 CTLs were treated with recombinant IFNα and IFNβ protein, respectively, for 1 h. Nuclear extracts were prepared from these cells and analyzed for STAT3 activation using EMSA with the WT pSTAT3 consensus probe (Santa Cruz Cat# sc-2571) and mutant probe (Santa Cruz Cat# sc-2572). Black arrow points to the DNA-pSTAT3 complex. **c**. Nuclear extracts were prepared as in B and analyzed for STAT3 activation using EMSA with the *Gzmb* promoter DNA probe GP4 as indicated in A. To determine pSTAT3-DNA binding specificity, the WT pSTAT3 consensus probe as shown in B was used for cold probe competition at the indicated ratios relative to the GP4 probe as a specificity control. Black arrow points to the DNA-pSTA T3 complex

STAT3 is a transcription factor. To determine whether STAT3 directly regulates *Gzmb* transcription, we then examined the *Gzmb* promoter and identified six putative STAT consensus sequence elements (Fig. 7A). Because STAT3 activation peaked at 1 h after IFNα and IFNβ stimulation (Fig. 6A), we treated the 2/20 CTLs with IFNα and IFNβ, respectively, for 1 h and analyzed protein-DNA interactions by EMSA. We firstly used the pSTAT3 consensus sequence probe as a positive control and the paired mutant probe as a negative control and observed that the activated STAT3 binds to the WT probe but not binding to the mutant probe (Fig. 7B). We then used the *Gzmb* promoter probes (Fig. 7A) and observed that IFNα- and IFNβ-induced STAT3 binds to the STAT consensus sequence element GP4 in the *Gzmb* promoter (Fig. 7C). Initial attempts failed to show anti-pSTAT3 antibody-dependent supershift. We used IL6-treated tumor cells as a positive control and also observed no supershift by anti-pSTAT3 antibody (Additional file 1 Figure S3). However, competition with the cold WT pSTAT3 consensus sequence probe as shown in Fig. 7B revealed a dose-dependent effect against the *Gzmb* promoter DNA probe (Fig. 7C). Taken together, our data indicate that IFNα and IFNβ induce STAT3 activation and the activated STAT3 binds to the *Gzmb* promoter to upregulate granzyme B transcription in CTLs.

## Discussion

One of the mechanisms underlying IFN-I function in tumor suppression is their autologous tumor suppressive activity [1]. Consistent with this notion, we observed that IFNAR1-KO mice are more sensitive to carcinogen-induced tumor development. However, we also observed that IFNAR1-sufficient tumor cells grow significantly faster in IFNAR1-KO mice and in mice with IFNAR1 deficiency only in T cells. Our findings thus indicate that IFN-I also acts through regulating T cell function to execute its antitumor activity, and extend IFN-I function in tumor suppression to T cells.

The mechanism underlying IFN-I function in regulating T cell function in anti-tumor immune response was largely elusive. Although it has been shown that IFN-I positively regulates T cell activation, clonal expansion, memory cell differentiation and survival [38–42], we found that IFN-I is not essential in regulating T cell activation and differentiation in tumor-bearing mice. More importantly, IFNAR1-deficient T cells responded to antigen to generate antigen-specific T cells as efficiently as WT T cells in vivo in the mixed WT and IFNAR1-KO BM chimera mice models. It is known that IFN-I regulates DC function [25, 43, 44] and DC is essential for antigen-specific T cell activation. In our mixed BM chimera mice models, the WT BM likely generate functional DCs and therefore let us to unmask the function of IFN-I in T cells activation directly. We reproducibly found that loss of IFN-I function in T cells does not impair T cell activation and generation of antigen-specific T cells in vivo.

IFN-I virtually can activate all STATs to regulate target gene expression depending on the cellular context [45]. IFNα and IFNβ can activate both STAT1 [46] and STAT4 [47, 48]. However, STAT1 represses whereas STAT4 activates IFNγ expression in T cells during a viral infection [47, 48]. In this study, we determined that IFNα and IFNβ selectively induce activation of STAT1 and STAT3 in CTLs in vitro. We further determined that IFN-I activated STAT3 directly bound to the *Gzmb* promoter and inhibiting pSTAT3 decreased IFN-I-induced *Gzmb* expression in T cells. Furthermore, IFNAR1-deficient tumor-infiltrating and antigen-specific CTLs exhibited diminished *Gzmb* expression. Taken together, we have determined that IFN-I regulates CTL effector function through activating the STAT3-granzyme B axis in anti-tumor immune response.

In human breast cancer patients, the autologous IFN-I signaling in tumor cells controls tumor cell response to chemotherapy [1]. In human melanoma patients, the intrinsic IFN-I signaling pathway is essential for tumor response to checkpoint inhibitor immunotherapy [19, 20]. Our findings indicate that IFN-I intrinsic signaling in T cells is also essential for expression of granzyme B and T cell function in colon carcinoma growth control in vivo. We have therefore extended IFN-I functions to T cell anti-tumor immune response. It is known that the impaired IFN signaling pathway in lymphocytes is a common immune defect in human cancer [49]. We observed here that the IFNAR1 is significantly down-regulated in both the colon carcinoma tissues and CTLs of colon cancer patients as compared to the normal colon tissues and CTLs of healthy donors, respectively. Therefore, human colon carcinoma may use down-regulating IFNAR1 in CTLs as a mechanism to impair CTL effector function to evade host cancer immunosurveillance. Our findings indicate that IFNAR1 is potentially a therapeutic target for boosting CTL effector function in human colon cancer immunotherapy.

## Conclusion

Our studies aimed at determining the role of IFN-I in CTL-mediated tumor suppression in the tumor microenvironment. Previous studies have established an essential role of autologous IFN-I signaling in tumor cell response to chemotherapy and immunotherapy. It becomes critically important to determine whether IFN-I plays a role in CTL function in the tumor suppression since the IFNAR1 is down-regulated on CTLs from human colon cancer patients. It is clear that under the conditions used in our studies IFN-I intrinsic signaling pathway plays a fundamental role in T cell-mediated tumor suppression in vivo. Reversal of immune suppression to activate CTLs is the principle action mechanism of current checkpoint inhibitor immunotherapy. Our data determined that IFN-I is not essential for CTL activation but rather is critical in regulation of key CTL effector granzyme B expression and tumor growth control in vivo. We provide evidence that human colon carcinoma cells may use down-regulation of IFNAR1 to impair CTL effector function to evade host cancer immunosurveillance. Therefore, targeting IFNAR1 down-regulation is potentially an effective approach to bypass both PD-L1-depedent and PD-L1-independent immune suppressions to directly activate CTL effector function to suppress colon carcinoma growth.

## Additional files

Additional file 1: **Figure S1**. Immune cell profiles in WT and IFNAR1-TKO mice. **Figure S2**. Scheme of creation of WT and IFNAR1-KO mixed BM chimera mice. **Figure S3**. IL6 activates pSTAT3 in colon carcinoma cells.**Table S1**. Antibodies. **Table S2**. Differentially expressed immune genes between WT and IFNAR1 KO tumor-infiltrating CD8^+^ T cells from MCA and MC38 tumor-bearing mice. **Table S3**. Differentially expressed immune genes between activated WT and IFNAR1 KO CD8^+^ T cells from mixed chimera mice. (PDF 506 kb)

## Data Availability

Data and material presented in this study are available upon request.

## Abbreviations

CTLs: Cytotoxic T lymphocytes
IFNAR1: Interferon (alpha and beta) receptor 1
IFN-I: Type I interferons
MCA: Methylcholanthrene

## References

[1] Sistigu A, Yamazaki T, Vacchelli E, Chaba K, Enot DP, Adam J (2014). Cancer cell-autonomous contribution of type I interferon signaling to the efficacy of chemotherapy. Nat Med.

[2] Stone ML, Chiappinelli KB, Li H, Murphy LM, Travers ME, Topper MJ (2017). Epigenetic therapy activates type I interferon signaling in murine ovarian cancer to reduce immunosuppression and tumor burden. Proc Natl Acad Sci U S A.

[3] Cauwels A, Van Lint S, Garcin G, Bultinck J, Paul F, Gerlo S (2018). A safe and highly efficient tumor-targeted type I interferon immunotherapy depends on the tumor microenvironment. Oncoimmunology..

[4] Cauwels A, Van Lint S, Paul F, Garcin G, De Koker S, Van Parys A (2018). Delivering type I interferon to dendritic cells empowers tumor eradication and immune combination treatments. Cancer Res.

[5] Brown Michael C., Holl Eda K., Boczkowski David, Dobrikova Elena, Mosaheb Mubeen, Chandramohan Vidya, Bigner Darell D., Gromeier Matthias, Nair Smita K. (2017). Cancer immunotherapy with recombinant poliovirus induces IFN-dominant activation of dendritic cells and tumor antigen–specific CTLs. Science Translational Medicine.

[6] Kirkwood J (2002). Cancer immunotherapy: the interferon-alpha experience. Semin Oncol.

[7] Garbe C, Eigentler TK (2007). Diagnosis and treatment of cutaneous melanoma: state of the art 2006. Melanoma Res.

[8] Hervas-Stubbs S, Perez-Gracia JL, Rouzaut A, Sanmamed MF, Le Bon A, Melero I (2011). Direct effects of type I interferons on cells of the immune system. Clin Cancer Res.

[9] Ayers M, Lunceford J, Nebozhyn M, Murphy E, Loboda A, Kaufman DR (2017). IFN-gamma-related mRNA profile predicts clinical response to PD-1 blockade. J Clin Invest.

[10] van Pesch V, Lanaya H, Renauld JC, Michiels T (2004). Characterization of the murine alpha interferon gene family. J Virol.

[11] Ivashkiv LB, Donlin LT (2014). Regulation of type I interferon responses. Nat Rev Immunol..

[12] Balkwill F, Watling D, Taylor-Papadimitriou J (1978). Inhibition by lymphoblastoid interferon of growth of cells derived from the human breast. Int J Cancer.

[13] Hobeika AC, Subramaniam PS, Johnson HM (1997). IFNalpha induces the expression of the cyclin-dependent kinase inhibitor p21 in human prostate cancer cells. Oncogene..

[14] Greiner JW, Hand PH, Noguchi P, Fisher PB, Pestka S, Schlom J (1984). Enhanced expression of surface tumor-associated antigens on human breast and colon tumor cells after recombinant human leukocyte alpha-interferon treatment. Cancer Res.

[15] Lu M, Zhang W, Li Y, Berenzon D, Wang X, Wang J (2010). Interferon-alpha targets JAK2V617F-positive hematopoietic progenitor cells and acts through the p38 MAPK pathway. Exp Hematol.

[16] Xiao W, Klement JD, Lu C, Ibrahim ML, Liu K (2018). IFNAR1 controls autocrine type I IFN regulation of PD-L1 expression in myeloid-derived suppressor cells. J Immunol.

[17] Chawla-Sarkar M, Lindner DJ, Liu YF, Williams BR, Sen GC, Silverman RH (2003). Apoptosis and interferons: role of interferon-stimulated genes as mediators of apoptosis. Apoptosis..

[18] Castiello L, Sestili P, Schiavoni G, Dattilo R, Monque DM, Ciaffoni F (2018). Disruption of IFN-I signaling promotes HER2/Neu tumor progression and breast Cancer stem cells. Cancer Immunol Res..

[19] Zaretsky JM, Garcia-Diaz A, Shin DS, Escuin-Ordinas H, Hugo W, Hu-Lieskovan S (2016). Mutations associated with acquired resistance to PD-1 blockade in melanoma. N Engl J Med.

[20] Shin DS, Zaretsky JM, Escuin-Ordinas H, Garcia-Diaz A, Hu-Lieskovan S, Kalbasi A (2017). Primary resistance to PD-1 blockade mediated by JAK1/2 mutations. Cancer Discov.

[21] Shankaran V, Ikeda H, Bruce AT, White JM, Swanson PE, Old LJ (2001). IFNgamma and lymphocytes prevent primary tumour development and shape tumour immunogenicity. Nature.

[22] Hanson HL, Donermeyer DL, Ikeda H, White JM, Shankaran V, Old LJ (2000). Eradication of established tumors by CD8+ T cell adoptive immunotherapy. Immunity.

[23] Leone RD, Sun IM, Oh MH, Sun IH, Wen J, Englert J, et al. Inhibition of the adenosine A2a receptor modulates expression of T cell coinhibitory receptors and improves effector function for enhanced checkpoint blockade and ACT in murine cancer models. Cancer Immunol Immunother. 2018. 10.1007/s00262-018-2186-0 PMC11028354 29923026

[24] Dunn GP, Bruce AT, Sheehan KC, Shankaran V, Uppaluri R, Bui JD (2005). A critical function for type I interferons in cancer immunoediting. Nat Immunol.

[25] Fuertes MB, Kacha AK, Kline J, Woo SR, Kranz DM, Murphy KM (2011). Host type I IFN signals are required for antitumor CD8+ T cell responses through CD8{alpha}+ dendritic cells. J Exp Med.

[26] Redd PS, Lu C, Klement JD, Ibrahim ML, Zhou G, Kumai T (2018). H3K4me3 mediates the NF-kappaB p50 homodimer binding to the pdcd1 promoter to activate PD-1 transcription in T cells. Oncoimmunology..

[27] Kumai T, Lee S, Cho HI, Sultan H, Kobayashi H, Harabuchi Y (2017). Optimization of peptide vaccines to induce robust antitumor CD4 T-cell responses. Cancer Immunol Res.

[28] Nagato T, Lee YR, Harabuchi Y, Celis E (2014). Combinatorial immunotherapy of polyinosinic-polycytidylic acid and blockade of programmed death-ligand 1 induce effective CD8 T-cell responses against established tumors. Clin Cancer Res.

[29] Hodge JW, Schlom J (1999). Comparative studies of a retrovirus versus a poxvirus vector in whole tumor-cell vaccines. Cancer Res.

[30] Frank DA, Mahajan S, Ritz J (1999). Fludarabine-induced immunosuppression is associated with inhibition of STAT1 signaling. Nat Med.

[31] Schust J, Sperl B, Hollis A, Mayer TU, Berg T (2006). Stattic: a small-molecule inhibitor of STAT3 activation and dimerization. Chem Biol.

[32] Yang D, Stewart TJ, Smith KK, Georgi D, Abrams SI, Liu K (2008). Downregulation of IFN-gammaR in association with loss of Fas function is linked to tumor progression. Int J Cancer.

[33] Lu C, Redd PS, Lee JR, Savage N, Liu K (2016). The expression profiles and regulation of PD-L1 in tumor-induced myeloid-derived suppressor cells. Oncoimmunology.

[34] Lu C, Yang D, Sabbatini ME, Colby AH, Grinstaff MW, Oberlies NH (2018). Contrasting roles of H3K4me3 and H3K9me3 in regulation of apoptosis and gemcitabine resistance in human pancreatic cancer cells. BMC Cancer.

[35] Golstein P, Griffiths GM. An early history of T cell-mediated cytotoxicity. Nat Rev Immunol. 2018. 10.1038/s41577-018-0009-3 29662120

[36] Gawden-Bone CM, Frazer GL, Richard AC, Ma CY, Strege K, Griffiths GM (2018). PIP5 kinases regulate membrane phosphoinositide and actin composition for targeted granule secretion by cytotoxic lymphocytes. Immunity..

[37] Ryan MH, Bristol JA, McDuffie E, Abrams SI (2001). Regression of extensive pulmonary metastases in mice by adoptive transfer of antigen-specific CD8(+) CTL reactive against tumor cells expressing a naturally occurring rejection epitope. J Immunol.

[38] Kolumam GA, Thomas S, Thompson LJ, Sprent J, Murali-Krishna K (2005). Type I interferons act directly on CD8 T cells to allow clonal expansion and memory formation in response to viral infection. J Exp Med.

[39] Marrack P, Kappler J, Mitchell T (1999). Type I interferons keep activated T cells alive. J Exp Med.

[40] Curtsinger JM, Valenzuela JO, Agarwal P, Lins D, Mescher MF (2005). Type I IFNs provide a third signal to CD8 T cells to stimulate clonal expansion and differentiation. J Immunol.

[41] Le Bon A, Durand V, Kamphuis E, Thompson C, Bulfone-Paus S, Rossmann C (2006). Direct stimulation of T cells by type I IFN enhances the CD8+ T cell response during cross-priming. J Immunol.

[42] Aichele P, Unsoeld H, Koschella M, Schweier O, Kalinke U, Vucikuja S (2006). CD8 T cells specific for lymphocytic choriomeningitis virus require type I IFN receptor for clonal expansion. J Immunol.

[43] Schiavoni G, Mattei F, Gabriele L (2013). Type I interferons as stimulators of DC-mediated cross-priming: impact on anti-tumor response. Front Immunol.

[44] Tzeng A, Kauke MJ, Zhu EF, Moynihan KD, Opel CF, Yang NJ (2016). Temporally programmed CD8alpha(+) DC activation enhances combination Cancer immunotherapy. Cell Rep.

[45] Zitvogel L, Galluzzi L, Kepp O, Smyth MJ, Kroemer G (2015). Type I interferons in anticancer immunity. Nat Rev Immunol.

[46] Catalfamo M, Wilhelm C, Tcheung L, Proschan M, Friesen T, Park JH (2011). CD4 and CD8 T cell immune activation during chronic HIV infection: roles of homeostasis, HIV, type I IFN, and IL-7. J Immunol.

[47] Nguyen KB, Cousens LP, Doughty LA, Pien GC, Durbin JE, Biron CA (2000). Interferon alpha/beta-mediated inhibition and promotion of interferon gamma: STAT1 resolves a paradox. Nat Immunol.

[48] Nguyen KB, Watford WT, Salomon R, Hofmann SR, Pien GC, Morinobu A (2002). Critical role for STAT4 activation by type 1 interferons in the interferon-gamma response to viral infection. Science.

[49] Critchley-Thorne RJ, Simons DL, Yan N, Miyahira AK, Dirbas FM, Johnson DL (2009). Impaired interferon signaling is a common immune defect in human cancer. Proc Natl Acad Sci U S A.

