# Supplementary material for: Type I interferon suppresses tumor growth through activating the STAT3-granzyme B pathway in tumor-infiltrating cytotoxic T lymphocytes
Source: J Immunother Cancer. 2019 Jun 22;7:157. doi: 10.1186/s40425-019-0635-8 (PMC6589175; doi:10.1186/s40425-019-0635-8)
Supplement: Supplementary file 1 — Figure S1. Immune cell profiles in WT and IFNAR1-TKO mice. Figure S2. Scheme of creation of WT and IFNAR1-KO mixed BM chimera mice. Figure S3. IL6 activates pSTAT3 in colon carcinoma cells.Table S1. Antibodies. Table S2. Differentially expressed immune genes between WT and IFNAR1 KO tumor-infiltrating CD8+ T cells from MCA and MC38 tumor-bearing mice. Table S3. Differentially expressed immune genes between activated WT and IFNAR1 KO CD8+ T cells from mixed chimera mice. (PDF 506 kb) [file 40425_2019_635_MOESM1_ESM.pdf]

Supplemental Data

1. Figure S1. Immune cell profiles in WT and IFNAR1-TKO mice.

2. Figure S2. Scheme of creation of WT and IFNAR1-KO mixed BM chimera mice.

3. Figure S3. IL6 activates pSTAT3 in colon carcinoma cells.

4. Table S1. Antibodies.

5. Table S2. Differentially expressed immune genes between WT and IFNAR1 KO tumor-infiltrating $CD8^+$ T cells from MCA and MC38 tumor-bearing mice.

6. Table S3. Differentially expressed immune genes between activated WT and IFNAR1 KO $CD8^+$ T cells from mixed chimera mice.

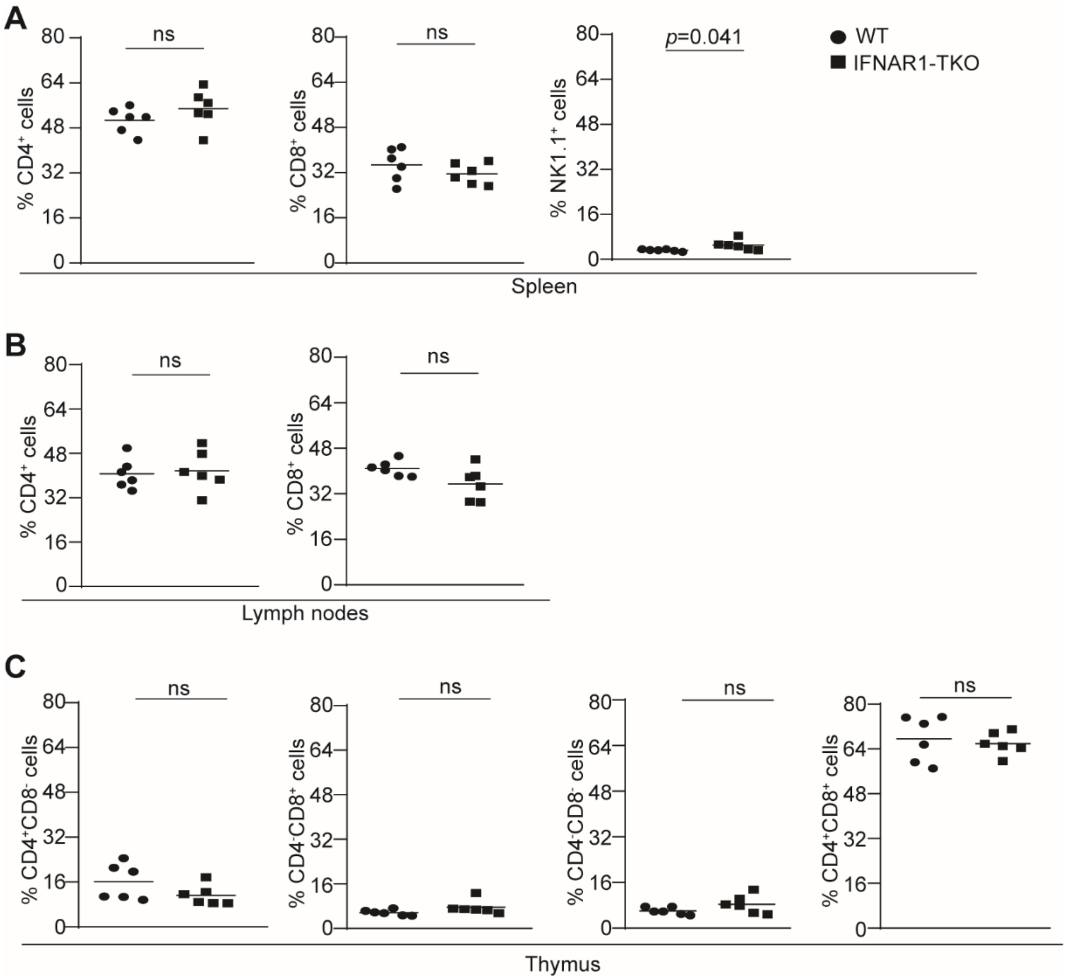

Figure S1. Immune cell profiles in WT and IFNAR1-TKO mice. A. Spleens were collected from WT C57BL/6 (n=6) and IFNAR1-TKO (n=6) mice, stained with CD4-, CD8-, and NK1.1-specific mAbs, and analyzed by flow cytometry. Shown are percentages of the indicated cells. **B**. Lymph nodes were collected from WT C57BL/6 (n=6) and IFNAR1-TKO (n=6) mice, stained with CD4-, and CD8-specific mAbs, and analyzed by flow cytometry. Shown are percentages of the indicated cells. **C**. Thymuses were collected from WT C57BL/6 (n=6) and IFNAR1-TKO (n=6) mice, stained with CD4- and CD8-specific mAbs, and analyzed by flow cytometry. Shown are percentages of the indicated cells.

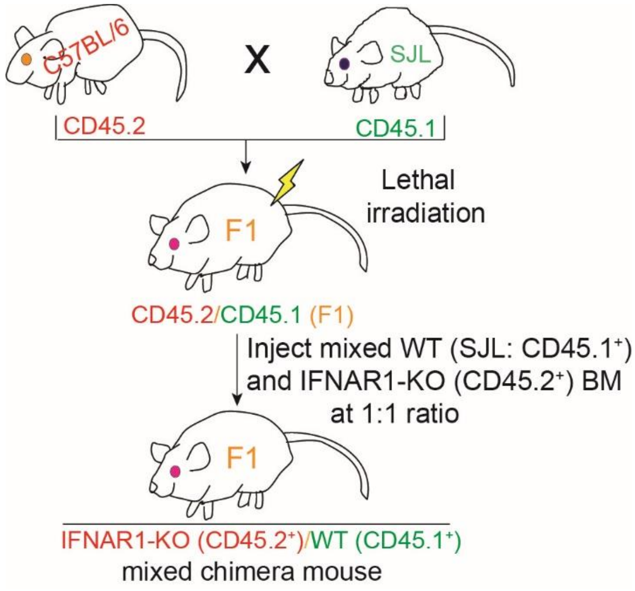

Figure S2. Scheme of creation of WT and IFNAR1-KO mixed BM chimera mice.

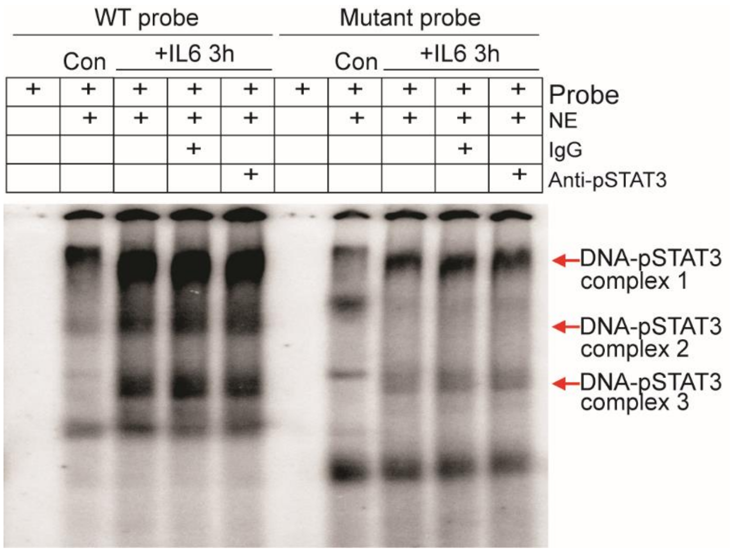

Figure S3. IL6 activates pSTAT3 in colon carcinoma cells. MC38 tumor cells were treated with IL6 for 3h. Nuclear extracts were prepared from the untreated (control) and treated cells and analyzed for STAT3 activation using EMSA with the WT pSTAT3 consensus probe (Santa Cruz Cat# sc-2571) and mutant probe (Santa Cruz Cat# sc-2572). Anti-pSTAT3 (Santa Cruz Cat#sc-8059X) were used to test supershift. Red arrows point to the DNA-pSTAT3 complexes.

Table S1. Antibodies

Antibody	Source	Clone	Cat Number	Application
FITC anti-mouse CD8	Biolegend	53-6.7	100706	Flow Cytometry
PerCP anti-mouse CD8	Biolegend	53-6.7	100732	Flow Cytometry
FITC anti-mouse CD4	Biolegend	RM4-5	100512	Flow Cytometry
PerCP anti-mouse CD4	Biolegend	RM4-5	100538	Flow Cytometry
FITC anti-mouse MHCII	Biolegend	M5/114.15.2	107606	Flow Cytometry
FITC anti-mouse CD45	Biolegend	30-F11	103108	Flow Cytometry
APC/Fire anti-mouse CD45.1	Biolegend	A20	110752	Flow Cytometry
PB anti-mouse CD45.2	Biolegend	104	109820	Flow Cytometry
FITC anti-mouse CD11b	Biolegend	M1/70	101206	Flow Cytometry
PE anti-mouse NK1.1	eBioscience	PK136	12-5941-82	Flow Cytometry
PerCP anti-mouse Gr1	Biolegend	RB6-8C5	108426	Flow Cytometry
FITC anti-human CD8	Biolegend	HIT8a	300906	Flow Cytometry
PE anti-human CD3	Biolegend	HIT3a	300308	Flow Cytometry
APC anti-huamn IFNAR	R&D	85228	FAB245A	Flow Cytometry
Zombie Violet	Biolegend		423114	Flow Cytometry
Zombie UV	Biolegend		423108	Flow Cytometry
MojoSort Mouse CD8 Nanobeads	Biolegend		480035	Cell isolation
Stattic	Santa Cruz		sc-202818	In vitro treatment
Fludarabine	Santa Cruz		sc-204755	In vitro treatment
pSTAT3	Santa Cruz	B-7	sc-8059X	EMSA
pSTAT1 (Y701)	BD transduction laboratory	14	612133	WB
STAT1 (C-terminus)	BD transduction laboratory	42	51-90002093	WB
pSTAT2 (Y690)	Abcam	polyclonal	ab53132	WB
STAT2	Santa cruz	H190	sc-22816	WB
pSTAT3 (Y705)	Upstate	9E12	05-485	WB
STAT3	BD transduction laboratory	84	610190	WB
pSTAT4 (Y693)	Santa cruz	pY693.38	sc-136194	WB
STAT4	Santa cruz	H119	sc-7959	WB
pSTAT5 (Y694)	BD Bioscience Pharmigen	47	558242	WB
STAT5	BD transduction laboratory	89	51-9002096	WB
pSTAT6 (Y641)	Santa cruz	pY641.18	sc-136019	WB
STAT6	BD transduction laboratory	23	51-9002196	WB
β-actin	Sigma	AC-15	A5441	WB

Table S2. Differentially expressed immune genes between WT and IFNAR1 KO tumor-infiltrating CD8$^+$ T cells from MCA and MC38 tumor-bearing mice

		MCA tumor							MC38 tumor						
		WT			KO			KO mean/WT mean	WT			KO			KO mean/WT mean
		WT1	WT2	Mean	KO1	KO2	Mean		WT1	WT2	Mean	KO1	KO2	Mean	
Gene	Accession #	820 CD8$^+$	821 CD8$^+$	**WT CD8$^+$**	393 CD8$^+$	394 CD8$^+$	**KO CD8$^+$**	KO vs WT	2425 CD8$^+$	398 CD8$^+$	**WT CD8$^+$**	801 CD8$^+$	805 CD8$^+$	**KO CD8$^+$**	KO vs WT
Aicda	NM_009645.2	1.25	1.52	1.385	1.54	8.22	4.88	3.523465704	38.9	23.35	31.125	5.11	16.51	10.81	0.347309237
Btla	NM_177584.3	178.18	146.98	162.58	59.9	84.49	72.195	0.44405831	48.37	47.4	47.885	22.22	21.48	21.85	0.456301556
C7	XM_356827.6	1.25	3.82	2.535	13.7	11.74	12.72	5.017751479	9.21	1.85	5.53	1	1	1	0.180831826
C8b	NM_133882.2	1.25	2.3	1.775	1.54	5.88	3.71	2.090140845	16.16	32.6	24.38	8.83	2.16	5.495	0.225389664
C9	NM_013485.1	1.25	11.44	6.345	18.32	7.05	12.685	1.999211978	16.16	36.3	26.23	6.6	2.16	4.38	0.166984369
Camp	NM_009921.2	1.25	1.52	1.385	1.54	49.29	25.415	18.35018051	17.42	4.86	11.14	38.58	40.81	39.695	3.563285458
Ccl12	NM_011331.2	841.6	1616.6	1229.1	127.66	933.91	530.785	0.431848507	1552.33	1580.77	1566.55	458.05	439.52	448.785	0.286479844
Ccl20	NM_016960.1	1.25	3.82	2.535	10.62	1.17	5.895	2.325443787	17.42	12.25	14.835	6.6	1	3.8	0.256150994
Ccl3	NM_011337.1	2174.68	3122.77	2648.725	374.06	715.69	544.875	0.205712182	943.42	618.94	781.18	210.38	200.4	205.39	0.262922758
Ccl4	NM_013652.1	926.55	1991.24	1458.895	72.22	252.26	162.24	0.111207455	421.04	372.94	396.99	71.31	60.14	65.725	0.165558326
Ccl7	NM_013654.2	757.89	5070.6	2914.245	292.44	903.4	597.92	0.205171494	2994.38	2476.01	2735.195	1195.84	1135.87	1165.855	0.426242005
Ccl8	NM_021443.2	446.79	2234.91	1340.85	56.82	529.14	292.98	0.218503188	1009.11	741.02	875.065	530.19	330.18	430.185	0.491603481
Ccr5	NM_009917.5	560.49	934.33	747.41	189.26	316.79	253.025	0.338535743	510.74	929.69	720.215	231.95	281.58	256.765	0.356511597
Ccr6	NM_001190333.1	19.51	8.39	13.95	1.54	9.4	5.47	0.392114695	26.89	32.6	29.745	6.6	6.02	6.31	0.212136494
Ccr7	NM_007719.2	1451.29	1418.62	1434.955	35.26	468.13	251.695	0.175402713	443.78	241.61	342.695	134.52	78.36	106.44	0.310596886
Ccr8	NM_007720.2	27.01	78.44	52.725	1.54	8.22	4.88	0.092555714	13	21.5	17.25	13.29	3.81	8.55	0.495652174
Ccr9	NM_009913.6	186.93	102.81	144.87	16.78	41.08	28.93	0.199696279	162.7	65.89	114.295	38.58	10.99	24.785	0.216851131
Ccrl2	NM_017466.4	680.43	2173.99	1427.21	196.96	667.58	432.27	0.302877642	566.32	371.09	468.705	141.22	145.73	143.475	0.306109387
Cd28	NM_007642.4	68.24	258.15	163.195	35.26	23.48	29.37	0.179968749	35.74	30.75	33.245	4.37	7.68	6.025	0.18123026
Cd4	NM_013488.2	115.71	238.35	177.03	86.08	84.49	85.285	0.481754505	85.64	49.25	67.445	17.76	12.65	15.205	0.225442954
Cd40lg	NM_011616.2	14.51	140.88	77.695	7.54	14.09	10.815	0.139198147	14.89	21.5	18.195	1	3.26	2.13	0.117065128
Cd69	NM_001033122.3	712.91	1503.9	1108.405	6	235.83	120.915	0.109089187	265.03	228.67	246.85	71.31	36.39	53.85	0.218148673
Cd79a	NM_007655.3	1.25	3.82	2.535	21.4	8.22	14.81	5.842209073	13.63	19.65	16.64	3.62	2.16	2.89	0.173677885
Cd79b	NM_008339.2	189.42	119.56	154.49	19.86	73.93	46.895	0.303547155	43.32	67.74	55.53	34.86	18.72	26.79	0.482441923
Cfb	NM_008198.2	363.09	742.44	552.765	19.86	103.26	61.56	0.111367398	367.98	676.28	522.13	254.27	239.06	246.665	0.472420662
Cr2	NM_007758.2	4.52	5.34	4.93	1.54	2.36	1.95	0.395537525	8.58	3.01	5.795	2.14	1	1.57	0.27092321
Ctla4	NM_009843.3	880.33	2454.21	1667.27	30.64	317.96	174.3	0.104542156	157.01	91.79	124.4	28.91	17.62	23.265	0.187017685
Ctss	NM_021281.2	8988.75	11520.19	10254.47	1686.15	4487.64	3086.895	0.30102921	2636.87	6354.75	4495.81	2417.8	1853.76	2135.78	0.475060112
Cxcl13	NM_018866.2	30.75	64.74	47.745	53.74	200.64	127.19	2.663943868	15.52	4.86	10.19	4.37	4.92	4.645	0.455839058
Cxcl9	NM_008599.2	1115.21	1266.33	1190.77	115.34	432.94	274.14	0.230220782	271.97	543.11	407.54	171.71	105.97	138.84	0.340678216
Cxcr1	NM_178241.4	1.25	2.3	1.775	9.08	1.17	5.125	2.887323944	27.53	15.95	21.74	1.39	9.33	5.36	0.246550138
Cxcr2	NM_009909.3	1.25	19.05	10.15	12.16	31.69	21.925	2.160098522	91.95	10.41	51.18	8.09	13.75	10.92	0.213364596
Cxcr5	NM_007551.2	34.5	43.42	38.96	1.54	4.71	3.125	0.080210472	25.63	14.1	19.865	5.11	3.81	4.46	0.224515479
Dpp4	NM_001159543.1	161.94	294.7	228.32	27.56	72.75	50.155	0.219669762	56.58	51.1	53.84	28.91	22.59	25.75	0.478268945
Fasl	NM_010177.3	105.72	424.15	264.935	7.54	43.42	25.48	0.096174533	42.68	28.9	35.79	10.32	16.51	13.415	0.37482537
Fcer1a	NM_010184.1	17.01	192.66	104.835	16.78	63.37	40.075	0.382267373	46.47	60.35	53.41	18.5	18.72	18.61	0.348436622
Fcer1g	NM_010185.4	1335.1	1109.47	1222.285	561.94	586.63	574.285	0.469845412	699.6	1053.62	876.61	413.43	364.97	389.2	0.443983071
Fcgr1	NM_010186.5	916.56	1197.8	1057.18	147.68	282.76	215.22	0.203579334	987	1262.63	1124.815	269.88	209.24	239.56	0.212977245
Fcgr4	NM_144559.1	1120.21	1281.56	1200.885	164.62	240.53	202.575	0.168688093	383.14	591.2	487.17	89.9	76.71	83.305	0.170997804
Folr4	NM_022888.2	35.75	31.23	33.49	6	8.22	7.11	0.21230218	20.58	21.5	21.04	1	1	1	0.047528517
Foxp3	NM_054039.1	42	159.16	100.58	15.24	15.26	15.25	0.151620601	26.26	27.05	26.655	4.37	7.68	6.025	0.226036391
Gfi1	NM_010278.2	24.51	63.22	43.865	6	22.3	14.15	0.322580645	18.68	25.2	21.94	1	10.44	5.72	0.26071103
Gzma	NM_010370.2	746.64	810.97	778.805	115.34	99.74	107.54	0.138083346	79.32	38.15	58.735	25.94	15.96	20.95	0.356686814

Gzmb	NM_013542.2	649.19	1394.25	1021.72	16.78	159.57	88.175	0.086300552	299.77	195.37	247.57	136.75	93.82	115.285	0.465666276
H2-Q10	NM_010391.4	28.26	110.43	69.345	39.88	29.34	34.61	0.499098709	63.53	28.9	46.215	11.81	10.44	11.125	0.240722709
Hamp	NM_032541.1	1.25	1.52	1.385	2.92	3.53	3.225	2.328519856	13.63	6.71	10.17	2.14	1	1.57	0.154375615
Icos	NM_017480.1	300.62	1298.31	799.465	13.7	138.45	76.075	0.095157387	112.8	89.94	101.37	26.68	15.41	21.045	0.207605801
Ifit2	NM_008332.2	1370.08	1634.88	1502.48	475.7	299.19	387.445	0.257870321	835.41	802.06	818.735	310.05	403.07	356.56	0.435501108
Ifna2	NM_010503.2	1.25	1.52	1.385	4.46	2.36	3.41	2.462093863	7.31	8.56	7.935	1	3.26	2.13	0.268431002
Ifng	NM_008337.1	194.42	495.73	345.075	7.54	69.23	38.385	0.111236688	35.11	32.6	33.855	19.24	6.02	12.63	0.373061586
Ikzf1	NM_001025597.1	175.68	305.36	240.52	95.32	58.67	76.995	0.320118909	86.27	113.99	100.13	41.56	51.86	46.71	0.466493558
Il10	NM_010548.1	14.51	139.36	76.935	6	25.82	15.91	0.206797946	17.42	12.25	14.835	1	1	1	0.067408156
Il10ra	NM_008348.2	890.32	1590.71	1240.515	252.4	409.47	330.935	0.266772268	403.36	526.46	464.91	236.42	206.48	221.45	0.476328752
Il11ra1	NM_010549.3	2.02	110.43	56.225	263.18	136.11	199.645	3.550822588	31.32	91.79	61.555	15.52	30.32	22.92	0.372349931
Il12b	NM_008352.1	50.74	46.46	48.6	1.54	8.22	4.88	0.100411523	34.47	14.1	24.285	8.09	4.92	6.505	0.267860819
Il17f	NM_145856.2	7.02	9.91	8.465	29.1	11.74	20.42	2.412285883	29.42	28.9	29.16	6.6	8.78	7.69	0.263717421
Il17re	NM_001034029.1	1.25	1.52	1.385	4.46	1.17	2.815	2.032490975	9.84	10.41	10.125	5.86	1.6	3.73	0.368395062
Il19	NM_001009940.1	1.25	2.3	1.775	22.94	8.22	15.58	8.777464789	37	30.75	33.875	8.83	6.02	7.425	0.219188192
Il1b	NM_008361.3	1518.76	2601.93	2060.345	281.66	1155.65	718.655	0.348803234	1353.36	655.94	1004.65	269.14	232.43	250.785	0.249624247
Il1rn	NM_031167.5	275.63	559.69	417.66	62.98	122.03	92.505	0.221483982	414.73	252.71	333.72	153.86	155.12	154.49	0.462932998
Il2	NM_008366.2	14.51	70.83	42.67	9.08	18.78	13.93	0.32645887	10.47	28.9	19.685	8.09	4.92	6.505	0.330454661
Il25	NM_080729.2	1.25	8.39	4.82	22.94	11.74	17.34	3.597510373	22.47	27.05	24.76	9.57	7.68	8.625	0.348344103
Il27	NM_145636.1	14.51	32.76	23.635	15.24	5.88	10.56	0.446795007	17.42	43.7	30.56	1	7.68	4.34	0.142015707
Il2ra	NM_008367.2	114.46	405.87	260.165	1.54	61.02	31.28	0.120231392	91.32	69.59	80.455	42.3	30.32	36.31	0.451308185
Il7r	NM_008372.3	279.38	801.83	540.605	143.06	289.8	216.43	0.400347759	166.49	184.27	175.38	59.41	50.75	55.08	0.314060896
Irf4	NM_013674.1	93.22	357.14	225.18	36.8	52.81	44.805	0.198974154	62.9	40	51.45	25.19	11.54	18.365	0.356948494
Kit	NM_001122733.1	28.26	174.39	101.325	47.58	432.94	240.26	2.371181841	15.52	8.56	12.04	25.94	23.69	24.815	2.061046512
Klra1	NM_016659.3	1.25	11.44	6.345	1.54	4.71	3.125	0.49251379	4.79	10.41	7.6	1	2.16	1.58	0.207894737
Klra7	NM_001110323.1	76.98	226.17	151.575	13.7	34.04	23.87	0.157479795	91.32	106.59	98.955	40.07	35.84	37.955	0.383558183
Klrc1	NM_001136068.1	1166.43	3364.92	2265.675	55.28	493.94	274.61	0.121204498	309.24	276.76	293	137.5	94.93	116.215	0.396638225
Klrc2	NM_001098669.1	33.25	108.9	71.075	13.7	18.78	16.24	0.228491031	12.37	15.95	14.16	2.14	4.36	3.25	0.229519774
Klrk1	NM_001083322.1	960.29	3249.18	2104.735	24.48	340.25	182.365	0.086645112	273.24	293.4	283.32	112.21	77.26	94.735	0.334374559
Masp1	NM_008555.2	170.68	1040.93	605.805	252.4	258.12	255.26	0.421356707	16.79	1.85	9.32	1.39	6.02	3.705	0.397532189
Pdcd1	NM_008798.1	625.45	1435.37	1030.41	42.96	463.44	253.2	0.245727429	108.38	173.18	140.78	53.46	44.12	48.79	0.346569115
Pla2g2a	NM_001082531.1	1.25	5.34	3.295	1.54	1.19	1.365	0.414264036	16.79	4.86	10.825	1	1	1	0.092378753
Pparg	NM_011146.1	15.76	41.89	28.825	86.08	192.42	139.25	4.830875976	33.21	28.9	31.055	7.34	7.13	7.235	0.232973756
Prf1	NM_011073.2	316.86	485.07	400.965	10.62	138.45	74.535	0.185889043	70.48	80.69	75.585	24.45	19.83	22.14	0.292915261
Psmb11	NM_175204.4	2.02	1.52	1.77	10.62	1.17	5.895	3.330508475	9.21	15.95	12.58	1	1	1	0.079491256
Ptgs2	NM_011198.3	1029	11166.87	6097.935	312.46	4397.3	2354.88	0.386176632	2310.94	1649.21	1980.075	927.35	916.08	921.715	0.465494994
S100a8	NM_013650.2	206.92	1411.01	808.965	720.56	3056.29	1888.425	2.334371697	2989.33	778.02	1883.675	692.33	1021.01	856.67	0.454786521
S100a9	NM_009114.2	195.67	809.45	502.56	538.84	2328.88	1433.86	2.853112066	2914.16	609.7	1761.93	505.65	747.66	626.655	0.355663959
Sele	NM_011345.2	32	999.82	515.91	39.88	479.87	259.875	0.503721579	11.1	10.41	10.755	2.14	3.26	2.7	0.251046025
Sell	NM_001164059.1	941.55	683.05	812.3	39.88	300.36	170.12	0.209430014	367.98	187.97	277.975	130.06	82.23	106.145	0.381850886
Slamf1	NM_013730.4	28.26	116.52	72.39	1.54	16.44	8.99	0.124188424	19.31	15.95	17.63	6.6	1	3.8	0.21554169
Slamf7	NM_144539.5	707.91	922.15	815.03	150.76	147.84	149.3	0.183183441	278.92	374.79	326.855	142.7	89.96	116.33	0.355907054
Stat4	NM_011487.4	95.72	172.87	134.295	1.54	36.38	18.96	0.141181727	47.74	30.75	39.245	19.99	11.54	15.765	0.401707224
Tagap	NM_145968.2	692.92	1808.49	1250.705	161.54	449.36	305.45	0.244222259	222.71	254.56	238.635	97.34	73.39	85.365	0.357722044
Tigit	NM_001146325.1	364.34	826.2	595.27	70.68	229.97	150.325	0.252532464	64.79	67.74	66.265	26.68	22.59	24.635	0.371764883
Tlr5	NM_016928.2	14.51	51.03	32.77	73.76	99.74	86.75	2.647238328	33.21	21.5	27.355	15.52	9.89	12.705	0.464448912
Tlr9	NM_031178.2	215.66	316.02	265.84	46.04	82.14	64.09	0.241084863	102.06	162.08	132.07	51.22	38.05	44.635	0.337964716
Tnf	NM_013693.1	74.48	296.22	185.35	10.62	41.08	25.85	0.139465875	136.8	163.93	150.365	58.66	64	61.33	0.407874173

Tnfaip3	NM_009397.2	2268.38	9053.05	5660.715	1293.45	3701.57	2497.51	0.441200449	873.3	670.73	772.015	308.56	275.51	292.035	0.378276329
Tnfrsf4	NM_011659.2	154.44	562.74	358.59	210.82	78.62	144.72	0.403580691	57.21	67.74	62.475	37.84	22.59	30.215	0.483633453
Tnfsf14	NM_019418.2	47	201.8	124.4	1.54	25.82	13.68	0.109967846	66.69	67.74	67.215	21.47	19.83	20.65	0.30722309
Tnfsf15	NM_177371.3	1.25	8.39	4.82	29.1	9.4	19.25	3.993775934	11.73	19.65	15.69	2.88	4.92	3.9	0.248565966
Tnfsf18	NM_183391.3	1.25	20.57	10.91	16.78	29.34	23.06	2.113657195	19.31	30.75	25.03	6.6	4.92	5.76	0.230123851
Tnfsf8	NM_009403.2	19.51	41.89	30.7	1.54	5.88	3.71	0.120846906	8.58	15.95	12.265	1	6.57	3.785	0.308601712
Tslp	NM_021367.1	22.01	16	19.005	78.38	29.34	53.86	2.833991055	6.68	3.01	4.845	1	1.6	1.3	0.268317853
Vtn	NM_011707.2	28.26	87.58	57.92	36.8	251.08	143.94	2.485151934	18.05	15.95	17	9.57	6.02	7.795	0.458529412
Xcr1	NM_011798.4	63.24	5.34	34.29	1.54	3.53	2.535	0.073928259	18.05	3.01	10.53	1	3.81	2.405	0.228395062

Table S3. Differentially expressed immune genes between activated WT and IFNAR1 KO CD8$^+$ T cells from mixed chimera mice

		WT			KO			KO mean/WT mean
Name	Accession#	CD8-WT1	CD8-WT2	WT mean	CD8-KO1	CD8-KO2	KO mean	
Abcb10	NM_019552.2	18	21	19.5	13	6	9.5	0.487179487
Aire	NM_009646.1	4	8	6	3	5	4	0.666666667
Blnk	NM_008528.4	87	71	79	36	30	33	0.417721519
Bst2	NM_198095.2	238	266	252	171	151	161	0.638888889
Btk	NM_013482.2	49	51	50	31	28	29.5	0.59
Btnl1	NM_001111094.1	9	15	12	3	6	4.5	0.375
C1qa	NM_007572.2	23	23	23	11	18	14.5	0.630434783
C1ra	NM_023143.3	11	16	13.5	6	8	7	0.518518519
C1s	NM_144938.2	26	31	28.5	11	9	10	0.350877193
C2	NM_013484.2	12	24	18	7	11	9	0.5
C3	NM_009778.2	14	22	18	30	26	28	1.555555556
C4bp	NM_007576.3	2	4	3	7	11	9	3
C6	NM_016704.2	15	20	17.5	12	7	9.5	0.542857143
C8b	NM_133882.2	18	23	20.5	13	14	13.5	0.658536585
C9	NM_013485.1	10	16	13	3	7	5	0.384615385
Card9	NM_001037747.1	20	17	18.5	36	24	30	1.621621622
Ccbp2	NM_021609.3	15	16	15.5	6	11	8.5	0.548387097
Ccl19	NM_011888.2	20	18	19	9	11	10	0.526315789
Ccl22	NM_009137.2	36	37	36.5	7	12	9.5	0.260273973
Ccl25	NM_009138.3	24	25	24.5	13	16	14.5	0.591836735
Ccl26	NM_001013412.2	6	6	6	4	3	3.5	0.583333333
Ccl3	NM_011337.1	767	829	798	504	412	458	0.573934837
Ccl6	NM_009139.2	32	39	35.5	73	74	73.5	2.070422535
Ccr10	NM_007721.4	14	23	18.5	13	10	11.5	0.621621622
Ccr6	NM_001190333.1	55	56	55.5	24	25	24.5	0.441441441
Ccrl1	NM_145700.2	9	12	10.5	3	3	3	0.285714286
Cd163	NM_053094.2	9	14	11.5	3	7	5	0.434782609
Cd19	NM_009844.2	251	243	247	130	95	112.5	0.455465587
Cd209g	NM_027343.3	12	9	10.5	4	7	5.5	0.523809524
Cd22	NM_001043317.2	108	130	119	63	48	55.5	0.466386555
Cd34	NM_001111059.1	25	24	24.5	10	11	10.5	0.428571429
Cd36	NM_007643.3	28	23	25.5	17	17	17	0.666666667
Cd40	NM_011611.2	37	37	37	17	19	18	0.486486486
Cd46	NM_010778.3	6	3	4.5	11	4	7.5	1.666666667
Cd55	NM_010016.2	310	312	311	234	174	204	0.655948553
Cd74	NM_001042605.1	2504	2691	2597.5	1240	1003	1121.5	0.431761309
Cd79a	NM_007655.3	11	17	14	5	3	4	0.285714286
Cd79b	NM_008339.2	385	381	383	229	183	206	0.537859008
Cd81	NM_133655.2	72	76	74	45	41	43	0.581081081
Cd83	NM_009856.2	97	141	119	69	64	66.5	0.558823529
Cfd	NM_013459.1	7	8	7.5	5	5	5	0.666666667
Cfh	NM_009888.3	15	17	16	8	7	7.5	0.46875
Cfi	NM_007686.2	5	4	4.5	1	3	2	0.444444444
Ciita	NM_007575.2	75	83	79	43	29	36	0.455696203

Clec4a4	NM_001005860.2	4	1	2.5	6	6	6	2.4
Clec4e	NM_019948.2	16	26	21	45	27	36	1.714285714
Clec5a	NM_001038604.1	9	9	9	27	14	20.5	2.277777778
Clu	NM_013492.2	7	18	12.5	5	7	6	0.48
Cr2	NM_007758.2	71	72	71.5	28	34	31	0.433566434
Csf2	NM_009969.4	13	13	13	2	5	3.5	0.269230769
Csf3r	NM_001252651.1	96	118	107	278	175	226.5	2.11682243
Ctsg	NM_007800.1	11	7	9	19	12	15.5	1.722222222
Cxcl10	NM_021274.1	23	27	25	7	16	11.5	0.46
Cxcl11	NM_019494.1	15	10	12.5	9	4	6.5	0.52
Cxcl13	NM_018866.2	6	9	7.5	2	7	4.5	0.6
Cxcl9	NM_008599.2	17	17	17	7	14	10.5	0.617647059
Cxcr2	NM_009909.3	49	54	51.5	121	112	116.5	2.262135922
Cxcr5	NM_007551.2	92	72	82	56	34	45	0.548780488
Cybb	NM_007807.2	101	119	110	76	69	72.5	0.659090909
Defb14	NM_183026.2	9	9	9	4	7	5.5	0.611111111
Fcamr	NM_001170632.1	15	19	17	9	11	10	0.588235294
Fcer1g	NM_010185.4	28	31	29.5	58	50	54	1.830508475
Fcgr1	NM_010186.5	10	12	11	8	4	6	0.545454545
Fcgr3	NM_010188.5	24	20	22	47	35	41	1.863636364
Fn1	NM_010233.1	33	29	31	17	18	17.5	0.564516129
Gzmb	NM_013542.2	498	544	521	373	299	336	0.644913628
H2-Aa	NM_010378.2	1559	1642	1600.5	733	547	640	0.399875039
H2-Ab1	NM_207105.2	802	844	823	340	273	306.5	0.372417983
H2-DMb2	NM_010388.4	270	300	285	129	109	119	0.41754386
H2-Eb1	NM_010382.2	179	215	197	77	73	75	0.38071066
H60a	NM_010400.2	8	9	8.5	4	5	4.5	0.529411765
Hamp	NM_032541.1	11	7	9	3	6	4.5	0.5
Hfe	NM_010424.4	21	26	23.5	12	15	13.5	0.574468085
Ifi204	NM_008329.2	13	25	19	9	12	10.5	0.552631579
Ifih1	NM_027835.2	134	128	131	68	48	58	0.442748092
Ifit2	NM_008332.2	125	149	137	79	57	68	0.496350365
Ifitm1	NM_001112715.1	84	94	89	181	159	170	1.91011236
Ifna1	NM_010502.2	44	55	49.5	23	24	23.5	0.474747475
Ifnb1	NM_010510.1	9	16	12.5	2	4	3	0.24
Ikzf2	NM_011770.4	105	93	99	69	63	66	0.666666667
Il10	NM_010548.1	9	10	9.5	8	4	6	0.631578947
Il12b	NM_008352.1	11	9	10	6	7	6.5	0.65
Il13ra1	NM_133990.4	17	26	21.5	38	31	34.5	1.604651163
Il17a	NM_010552.3	4	6	5	19	9	14	2.8
Il19	NM_001009940.1	6	10	8	5	5	5	0.625
Il1b	NM_008361.3	186	226	206	570	480	525	2.548543689
Il1r2	NM_010555.4	22	23	22.5	46	38	42	1.866666667
Il1rn	NM_031167.5	17	22	19.5	51	35	43	2.205128205
Il23a	NM_031252.1	10	17	13.5	9	3	6	0.444444444
Il23r	NM_144548.1	18	19	18.5	4	14	9	0.486486486
Il25	NM_080729.2	20	21	20.5	11	8	9.5	0.463414634
Il27	NM_145636.1	7	12	9.5	4	8	6	0.631578947

Il28a	NM_001024673.2	24	22	23	7	15	11	0.47826087
Il5	NM_010558.1	5	7	6	4	1	2.5	0.416666667
Irf5	NM_012057.3	47	54	50.5	29	34	31.5	0.623762376
Irf7	NM_016850.2	189	204	196.5	95	80	87.5	0.445292621
Irf8	NM_008320.3	66	69	67.5	33	37	35	0.518518519
Itgam	NM_001082960.1	53	50	51.5	96	67	81.5	1.582524272
Kir3dl1	NM_177749.3	5	6	5.5	3	2	2.5	0.454545455
Kir3dl2	NM_177748.2	13	10	11.5	5	7	6	0.52173913
Kit	NM_001122733.1	7	9	8	3	4	3.5	0.4375
Klra1	NM_016659.3	14	22	18	6	12	9	0.5
Klra21	NM_053151.1	4	5	4.5	2	3	2.5	0.555555556
Klra4	NM_010649.3	13	6	9.5	3	3	3	0.315789474
Klra5	NM_008463.2	13	15	14	5	3	4	0.285714286
Klra6	NM_008464.2	11	8	9.5	7	5	6	0.631578947
Klrc3	NM_021378.1	11	9	10	4	8	6	0.6
Lair1	NM_001113474.1	60	70	65	51	33	42	0.646153846
Ltb4r2	NM_020490.2	13	13	13	7	9	8	0.615384615
Ly86	NM_010745.2	49	80	64.5	40	34	37	0.573643411
Masp1	NM_008555.2	12	8	10	3	6	4.5	0.45
Mbl2	NM_010776.1	4	5	4.5	8	6	7	1.555555556
Mme	NM_008604.3	17	17	17	6	6	6	0.352941176
Ms4a1	NM_007641.5	376	410	393	219	165	192	0.488549618
Msr1	NM_001113326.1	14	13	13.5	2	10	6	0.444444444
Mx1	NM_010846.1	295	319	307	143	176	159.5	0.519543974
Nox1	NM_172203.1	11	11	11	5	8	6.5	0.590909091
Nox3	NM_198958.2	12	10	11	5	5	5	0.454545455
Nox4	NM_015760.4	2	7	4.5	2	2	2	0.444444444
Pax5	NM_008782.2	124	151	137.5	62	59	60.5	0.44
Pdcd1	NM_008798.1	218	256	237	148	148	148	0.624472574
Pdcd1lg2	NM_021396.2	25	34	29.5	21	14	17.5	0.593220339
Pdgfrb	NM_008809.1	11	13	12	3	7	5	0.416666667
Pigr	NM_011082.3	11	19	15	9	7	8	0.533333333
Pla2g2a	NM_001082531.1	20	27	23.5	9	5	7	0.29787234
Pla2g2e	NM_012044.2	18	15	16.5	7	4	5.5	0.333333333
Plau	NM_008873.2	12	15	13.5	5	6	5.5	0.407407407
Pml	NM_008884.2	273	328	300.5	207	151	179	0.595673877
Ptgs2	NM_011198.3	46	55	50.5	107	99	103	2.03960396
Rag1	NM_009019.2	6	8	7	1	8	4.5	0.642857143
Rag2	NM_009020.3	12	13	12.5	7	7	7	0.56
Rorc	NM_011281.2	20	13	16.5	6	7	6.5	0.393939394
S100a8	NM_013650.2	2788	3112	2950	6767	5402	6084.5	2.062542373
S100a9	NM_009114.2	2658	3017	2837.5	6693	5403	6048	2.131453744
Sele	NM_011345.2	9	8	8.5	1	6	3.5	0.411764706
Src	NM_001025395.2	10	25	17.5	8	11	9.5	0.542857143
Tal1	NM_011527.2	24	21	22.5	14	15	14.5	0.644444444
Tcf4	NM_013685.1	79	96	87.5	50	55	52.5	0.6
Tgfb2	NM_009367.1	2	3	2.5	7	4	5.5	2.2
Tgfbi	NM_009369.4	50	38	44	104	85	94.5	2.147727273

Tigit	NM_001146325.1	102	102	102	68	58	63	0.617647059
Tnfrsf13c	NM_028075.2	30	38	34	14	26	20	0.588235294
Tnfrsf17	NM_011608.1	11	14	12.5	5	8	6.5	0.52
Tnfsf12	NM_011614.3	12	25	18.5	9	8	8.5	0.459459459
Tnfsf15	NM_177371.3	10	6	8	5	5	5	0.625
Trem1	NM_021406.3	40	38	39	65	64	64.5	1.653846154
Tyrobp	NM_011662.2	150	171	160.5	383	300	341.5	2.127725857
Xcr1	NM_011798.4	8	10	9	5	4	4.5	0.5
